# Chloroform Inhalations

**Published:** 1853-10

**Authors:** 


*Chloroform Inhalations.*—A writer over the signature of G., in the Peninsular Journal of Medicine, says of the safety of chloroform inhalation, "we consider it about as safe to the patient as a journey by railroad or steamboat to the passengers. Our rule is never to use it in trivial cases, when the operation requires but a single stroke of the knife, or actually recommend it in any case. We make to our patients the above statement of its comparative safety, and if they elect, we administer it, taking due care that it is well mixed with atmospheric air, watching closely the pulse and respiration. With these cautions, we bide our time, await our turn for an accident, and confess to a growing dread of the agent."

A better illustration could not have been given, and inasmuch as its use is liable to be attended with fatal consequences, we do not think so powerful an agent should be administered, except in protracted surgical operations.

